# Problematic Attachment to Social Media: Five Behavioural Archetypes

**DOI:** 10.3390/ijerph16122136

**Published:** 2019-06-17

**Authors:** Majid Altuwairiqi, Nan Jiang, Raian Ali

**Affiliations:** Department of Computing and Informatics, Faculty of Science and Technology, Bournemouth University, Fern Barrow, Poole BH12 5BB, UK; njiang@bournemouth.ac.uk (N.J.); rali@bournemouth.ac.uk (R.A.)

**Keywords:** problematic attachment, behavioural archetypes, digital wellbeing, digital addiction, problematic online behaviour

## Abstract

Today, social media play an important role in people’s daily lives. Many people use social media to satisfy their personal and social needs, such as enhancing self-image, acquiring self-esteem, and gaining popularity. However, when social media are used obsessively and excessively, behavioural addiction symptoms can occur, leading to negative impacts on one’s life, which is defined as a problematic attachment to social media. Research suggests that tools can be provided to assist the change of problematic attachment behaviour, but it remains unclear how such tools should be designed and personalised to meet individual needs and profiles. This study makes the first attempt to tackle this problem by developing five behavioural archetypes, characterising how social media users differ in their problematic attachments to them. The archetypes are meant to facilitate effective ideation, creativity, and communication during the design process and helping the elicitation and customisation of the variability in the requirements and design of behaviour change tools for combatting problematic usage of social media. This was achieved by using a four-phase qualitative study where the diary study method was considered at the initial stage, and also the refinement and confirmation stage, to enhance ecological validity.

## 1. Introduction

Today, social media play an important role in people’s lives, as the use of social media has created a new set of social norms when individuals try to create and maintain an online persona that complements their physical presence [1]. Many people rely on social media to satisfy their personal and social needs, such as enhancing their self-esteem [2] and maximising their social capital [3]. Such reliance can become problematic (i.e., over-reliant), leading to negative impacts on one’s wellbeing due to the peer pressure and lower self-esteem as a result of comparing themselves unfavourably to others or believing that someone’s online material is always a true reflection of reality [4]. Moreover, the over-reliance on social media can result in the obsessive and excessive use of the media, which is often associated with undesirable life experiences, such as reduced creativity, increased anxiety, and a neglect of the reality of life [5,6]. Despite increasing awareness of the possible negative effects of excessive Internet use, certain individuals still seem to have strong feelings about, and intimate engagement with, digital devices and tend to ignore the associated risks.

However, like excessive and pathological Internet use [7,8,9,10], which is often described using different terms, such as Internet addiction, digital addiction, and cyber addiction [11,12,13,14,15,16], there is no agreed term yet to describe similar phenomena in the use of social media. In this paper, we consider the high penetration rate of social media and the similar risks between high social media exposure and problematic use of the Internet [17]. We use the term “problematic attachment to social media” instead of “social media addiction” to describ the usage style associated with a set of harmful consequences, such as negative emotions, destructive psychological states, and over-dependence. Originating from attachment theory [18], here attachment is used to denote an interaction style between users and the social media when the former overly rely on the latter to satisfy their social needs for relatedness and popularity with gratification while interacting with others online [19].

Recent studies have discovered similarities between the symptoms of social media use and those of classic addiction [20,21]. These symptoms include withdrawal (feeling anxiety when unable to connect as desired), tolerance (increasing online presence, interaction and accounts), relapse (after attempting to minimize or adjust one’s current usage habits), conflict (using social media despite having other priorities), and mood modification (feeling better when receiving likes and comments). Moreover, when people disconnect from or are asked to spend less time on social media and online interactions than desired, they become anxious, despite the lack of a clear and justified purpose for that online presence [22]. A report conducted in the United Kingdom shows that approximately 15 million Internet users (about 34% of the national population of Internet users) attempted a “digital detox” in 2016 [23]. However, when they went offline, 33% of participants reported having an increased feeling about productivity, 27% felt a sense of liberation, and 25% reported that they were enjoying life more. The report also noted that 16% of participants had a strong fear of missing out (FoMO), 15% felt lost, and 14% felt neglected.

Some argue that interventions should be proposed to help people take control of their social media usage, e.g., [24,25], and more importantly, social media should be designed to accommodate diverse techniques for the self-regulation of problematic attachment styles to improve digital wellbeing [14]. However, it remains unclear how such an intervention method should be designed when a relationship with social media can exhibit intimacy and become a second nature for users to satisfy their social-emotional needs [26]. Most research on social media addiction, online identity, and online attachment only relied on offline data collection methods, which are subject to recall bias and limitations on ecological validity. For example, interviews [27,28], surveys [29,30], and focus groups [31] were used to capture hindsight feelings and experiences from participants without considering their as-is experiences to do with as-is scenarios.

In this paper, we report our work on reinvestigating users’ problematic relationships with social media from a longitudinal and temporal perspective. We argue that using methods like diary studies [32] is crucial to understanding the problematic attachment to social media, as these methods allow us to capture participants’ as-is experience (i.e., when that experience is happening) rather than asking them to recall the hindsight experience from a retrospective perspective. The impacts of our work are three-fold. First, this study takes a unique approach to enhance ecological validity by using a live method reinforced through a multi-phase qualitative research method which would benefit similar research in the future. Second, it makes the first attempt to tackle the problem using diary studies, which focus on capturing the as-is experience in association with the as-is problematic attachment scenarios. Third, it develops five user behavioural archetypes, which are based on the internal characteristics of the users, the emotions and the psychological states accompanying their social media experiences. These archetypes not only form a solid foundation for creating personas for the intervention tools but also help facilitate effective communication, ideation, and creativity during the design process.

## 2. Theoretical Framing

The following sections present findings from a review of traditional literature. This review was carried out to explore problematic attachment to social media, implement behavioural archetypes, and get in-depth insights from existing research to guide the analysis and findings discussion presented in this paper. We review the literature around users’ modelling and personas, the personal and social factors in problematic attachments to social media, and the use of behavioural archetypes in this area.

### 2.1. Use of Personas and Behavioural Archetypes in User Experience (UX) Design

The management of problematic attachment is a crucial step in informing how the development of social media could be affecting the behaviour of end users. This information is useful for developers who want to improve their design to take into account the behaviour of end users, while system development emphasises the benefits of employing human-centred design (HCD). Users can be represented through both segmentation and user modelling methods, such as archetypes and personas describing the characteristics, behaviour and needs of users.

Cooper [33] made an initial introduction to the concept of the Persona in the Human-Computer Interaction (HCI) community, which defined a persona as “a precise description of a user’s characteristics and what he/she wants to accomplish”. The persona is considered a fictionalised representation of a hypothetical group of users, based on the needs, demographics, and goals of that group. The biographical characteristics of a persona are used to guide design decisions and help a project team to visualise user segments effectively, thus enhancing their required solutions. However, the behaviour and further characteristics of users about system interaction may not always prove compatible. In the majority of cases, personas do not include emotional and psychological states or behavioural patterns, verifying effective interaction with social media [34,35]. On the other hand, behavioural archetypes capture patterns and thus facilitate the representation of system users from a behavioural perspective, including emotions and psychological states [36,37]. Archetypes were used in this study to assist in identifying problematic online attachment in terms of interaction design, thus providing developers with a viable model for validating user flows and interactive elements. User modelling is employed in HCI systems to improve both the user experience and the design of the system.

### 2.2. Personas and Sociability 

This section discusses the relationship between personas and sociability. Researchers have shown that a persona set that helps the success of a project tends to have four main features: They are (1) fictional, (2) engaging, (3) goal-directed, and (4) role-based [38,39]. In addition, Floyd et al. [34] also noted a number of types of personas: (1) quantitative, data-driven personas (obtained from inherent groupings in the quantitative data); (2) user archetypes (considered similar to personas, but more generic and defined by position or role); and (3) marketing personas (i.e., generated for marketing purposes). Engaging personas can be developed from research that gives insight into the social and cultural background of users. In addition, fictitious information can be used to balance the collected data and give life and more engaging representation. 

Personas contribute towards sociability representation and inclusivity in the technology design through the identification of specific groups, such as customers, capable of freely interacting under different circumstances, including taking part in social interaction. Personas, therefore, play a role in achieving the goals and desires of end users, addressing limitations, and providing guidelines for decision-making in the interaction space, i.e., websites.

Contextual sociology facilitates the creation of personas by capturing descriptions of the surrounding environment of users, along with their behavioural patterns, attitudes, skills, and goals. The personas thus generated can also be employed by sales departments to address common behavioural needs and the potential objections of given personas [40].

### 2.3. Developing Behavioural Archetypes for People with Problematic Online Attachment

People with problematic online attachment have difficulty managing their usage and maintaining aspects of their offline life, such as work, academic performance, and friendships. They are often vulnerable to loneliness and anxious feelings. Furthermore, people with problematic online attachment are characterised by a denial of the nature of their online behaviour, adverse reactions when they feel their freedom to continue using social media is being limited by reminders and timers, and relapse when they try to regulate their usage of social media and reduce their preoccupation with them [41,42,43]. Any intervention designed to address problematic online attachment needs to consider the differences in the characteristics, personality, and problematic style of users, as a different type of user may require different interventions to meet their specific needs for social wellness.

Developing behavioural archetypes and related scenarios will allow an intervention team to tailor the development of the intervention so that it meets the needs of each archetype [44,45,46,47]. Behavioural archetype-based interventions can help with the tailoring of motivational and coping strategies to facilitate the behavioural change process.

### 2.4. Personality in Problematic Attachment on Social Media

In social media practices, potential online users are divided into segments for online marketing. On the other hand, research studies have linked personality characteristics and social media usage from various perspectives, including romantic relationships and self-presentation in relation to the Internet [48,49]. Further studies have revealed that the narcissistic type of individual uses Facebook particularly frequently [50,51]. Researchers have also concluded that this trend is facilitated by Facebook encouraging its users to engage in self-promotion and superficial behaviours, i.e., posting photographs and making status updates. Moreover, a considerable volume of previously published studies has highlighted the role of personality in the use of social media in general [52,53,54], as well as Facebook specifically.

Most previous research in this field has focused on broad models of personality, of which the most frequently employed is the five-factor model, commonly known as the ‘Big Five’ [55]. The Big Five model is based on the concept that the personality of an individual can be described in terms of scores for the following five factors: conscientiousness; extraversion; openness to experience; agreeableness; and neuroticism. There are more detailed descriptions of these Big Five factors referring to a range of personality traits, i.e., individuals who are open to experience tend to be creative, curious and original, whereas those lacking such openness are generally rather more down to earth and conventional and have a narrow range of interests [56].

The research in [57] and [58] have investigated the relationship between Facebook usage and the Big Five, concluding that most factors are related to specific patterns, i.e., in comparison with introverts, extraverted individuals generally have more friends on Facebook and belong to more Facebook groups. Also, individuals demonstrating high neuroticism are more likely than emotionally stable individuals to prefer using the Wall, i.e., the online bulletin board for posts and comments among contacts. This is because the Wall feature allows people with neurotic tendencies to take their time formulating messages and responses, which reduces the potential for any unintentional revelation of their internal personality characteristics [57].

## 3. Behavioural Archetypes Creation and Validation: Our Research Method

**Figure 1:**
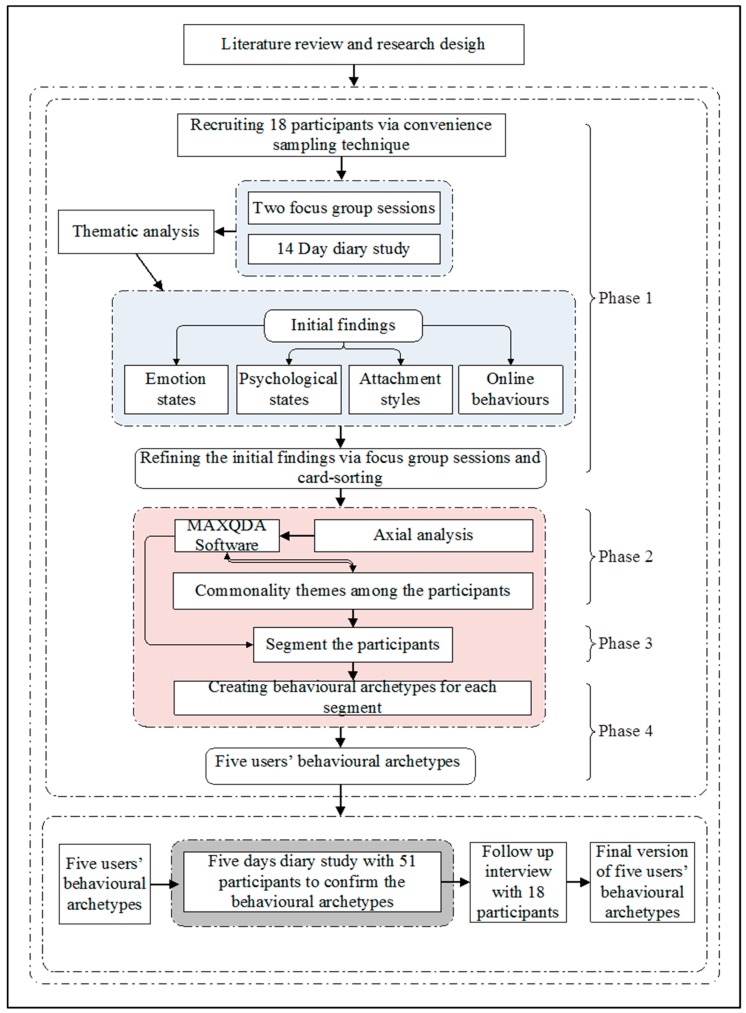
Overview of our research method and segmentation process.

The method we used to develop behavioural archetypes consisted of four phases, adapted from the traditional method of creating personas [59]. The first phase consisted of exploring and gathering qualitative data on users with problematic attachment to social media. The qualitative data emerging from the exploratory methods, such as focus groups and diary studies, were subjected to thematic analysis [60]. In phase two, axial coding was applied to the themes and categories that emerge from phase one in order to define patterns and commonalities and thus segment the participants. Then, in phase three, behavioural archetypes were created for each segment. Finally, in phase four, five high-level behavioural archetypes were developed and validated through a diary study. Figure 1 shows an overview of the research design. 

We also reviewed the relevant psychological literature: research on attachment theory [18,61], a cognitive behavioural model of pathological Internet use [62], Nomophobia [63], and Internet and Facebook addiction [9,64], as well as theories related to identity development, formation and affiliation i.e., identity theory [65,66], social identity theory [67], self-presentation theory [68,69], social role theory [70] and self-concept theory [71]. This review revealed a considerable number of sociological and psychological concepts related to problematic online attachment and helped us to develop hypotheses about social media users that were capable of filling the gap between our conceptions and the data-driven behavioural archetypes of users.

To obtain more insight into the four phases conducted to create behavioural archetypes are described in the following subsections of the current section. Then, the findings are described in Section 4, qualitative feedback on the behavioural archetypes are described in Section 5, and quantitative validation is described in Section 6. All the studies were approved by the research ethics committee of the authors’ institution.

### 3.1. First Phase: Qualitative Study

The aim of this study was to explore people’s problematic experiences and attachment to social media. A qualitative approach was adopted, consisting of focus groups and a diary study. The diary study was the core method of data collection in this phase. Focus groups were used to gather preliminary information that was elaborated via the diary study, which allowed us to capture detailed information about users’ problematic experience on a daily basis.

Eighteen participants were recruited via convenience sampling. Participants aged 18 years and over, and equal numbers of men and women were recruited. The eligibility criteria were (1) being an adult social media user and (2) being a self-declared problematic attachment to social media. A screening questionnaire was used to identify individuals with a problematic relationship with social media. The questionnaire was adapted from the Generalized Problematic Internet Use Scale [72]. We modified the phrasing to refer to “social media” rather than “the Internet” in general.

The first phase consisted of two focus group sessions, each with nine participants. These sessions aimed to familiarise the participants with the objective of the study and get insight into their problematic attachment to social media. At the end of the sessions, in preparation for the diary study, participants were trained to use the Evernote application. Evernote was chosen because it allows users to take notes, pictures, and recordings and share them with the research team on a daily basis. The collaborative feature of the application also allows a facilitator to send questionnaires to other users. The sample for the diary study consisted of the same 18 participants and lasted two weeks, during which participants completed two tasks. The first was to describe their online experience and online behaviour, with an emphasis on social media profiling features and online presence. In the second task, the participants were asked to forgo a specific social media activity, for example, to avoid replying instantly to friends’ messages, and to document the effects of doing so. Each participant chose which activity to forgo. The participants had to make notes three times a day (morning, afternoon and night) using the Evernote application. Daily reminders were also sent through the application. Once the diary study was completed, participants were invited to a face to face interview to go through their diary entries.

The data collected in the first phase were analysed in two iterations (the first iteration was carried out in phase one and the second in phase two). The first iteration used open coding based on a thematic analysis, to break down the data and identify first-level categories. The conceptual framework for the thematic analysis was based on the theoretical position of Braun and Clarke [60]. The findings were also validated through card sorting. See Appendix A for more details on the studies and the analysis results carried out in the first phase. Part of the result is published in [73,74].

### 3.2. Second Phase: Segmentation of Participants Based on the Qualitative Data 

**Figure 2:**
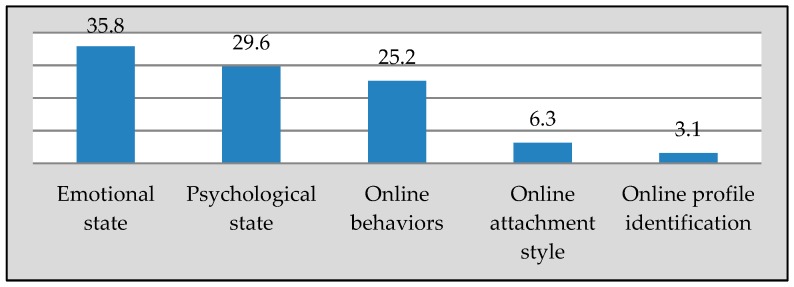
Common themes among all participants.

Segmentation involves taking several data points, as well as creating different groups based on commonalities shared by the members of a given group. The main goal in the development of the behavioural archetypes was to establish patterns and grouping similar individuals together. The second iteration in the analysis, which involved using axial coding, helped to generate high-level patterns and relationships between the categories that had emerged from the first iteration. As noted in Appendix A, data analysis was carried out with statistical analysis software (VERBI Software GmbH, Berlin, Germany). This second iteration in the analysis relied on the functions of the analysis software that enable a researcher to examine raw data, in order to elicit patterns and the existing commonalities in the extracted themes and sub-themes among all the participants (see Figure 2 below).

There are two qualitative methods of segmenting a population: one dimensional, such as by goals or usage lifecycle; and two dimensionally, such as by a combination of attitudes and behaviours [59]. Segmentation is a complex and challenging process. We developed the following questions to narrow down the segmentation options:

- What attachment style has contributed to the participant’s problematic usage?
- What are the main features of the participant’s online behaviour that could be classed as problematic?
- What is the participant’s identification for online interaction?
- What online behaviours have contributed to the participant’s problematic usage?

Understanding the data and commonalities led to the conclusion that the themes (variables) in Figure 2 were common to all the participants. The occurrence percentages varied between commonality variables due to the varying number of questions related to each variable within the study. After iterative revision and examination of the data and the responses to above questions, we found that considering a combination of online attachment styles and online behaviour variables can result in critical differences among the participants and, ultimately, better segmentation. In addition, after confirming the findings from the first phase, it became clear that the two dimensions used in the segmentation process were valid. Table 1 below illustrates the segmentation dimensions and processes. Segment one consists of six participants, segment two consists of four participants, segments three and four consist of three participants, and segment five consists of two participants.

In this table, the Y-axis is independent and represents the online behaviour dimension, comprising (1) kindness and self-presentation, (2) tracking information, (3) self-categorisation, (4) irresistible urges and self-disclosure, and (5) self-enhancement. The online attachment style dimension is represented by the X-axis (secure; FoMO; avoidant). Each segment is attained from the interaction between the Y-axis and X-axis. For example, segment one is attained from the intersection between the Y-axis (online behaviours) and X-axis (inline attachment style) and provides the first archetype (segment one).

### 3.3. Third Phase: Creating a Behavioural Archetype for Each Segment

In general, behavioural patterns, skills, and attitudes towards certain software packages, as well as fictional demographic or personal details, are intended to add depth to characters and make them seem more realistic. However, in the field of computations, such as HCI, there is insufficient consensus on the specific information required to establish a persona, including the presentation of information and the usage impacting the software design process. Goodwin [75] suggested that a researcher should, during the creation of such a persona, prioritise specifying important information pertinent to the design, i.e., the persona’s behaviour, workflow, goals, and attitudes and then add demographic and personal information. Such information is usually based on the assumptions of the designers and behaviour support systems specialists [76].

This research refines these components and proposes the building of constructs pertaining to the generation of behavioural archetypes obtained from the process of segmentation. These components provide additional details on the characteristics of users commonalities, as outlined in Figure 2. These commonalities include online profile identification, e.g., name and profile picture, online behaviours, e.g., role and motivation and feelings (emotional and psychological states). Behavioural archetypes do not include personal or demographic information, as such variables are not always related to behavioural patterns or the ways in which individuals interact with social media. The use of behavioural archetypes could assist in eliminating the gap between designers and behaviour analysts on the one hand, and users on the others, through appropriate consideration of the behavioural models of different groups of users, including how the design team can expect them to interact with the system. Furthermore, there should not be artificial restrictions on the content of behavioural archetypes; designers can enrich them with additional components, i.e., demographic information. 

The segmentation process led to the identification of five major behavioural archetypes in relation to the problematic use of social media: (1) the Secure behavioural archetype, (2) the Intimate behavioural archetype, (3) the Escapist behavioural archetype, (4) the Narcissist behavioural archetype, and (5) the Discrepancy behavioural archetype. Table 2 below presents these behavioural archetypes.

### 3.4. Fourth Phase: Developing and Validating Behavioural Archetypes

The findings resulting from previous phases and our earlier studies of behaviours associated with problematic attachment to social media [73,74] enabled the researchers to assess, validate, and refine the five main behavioural archetypes, i.e., the Secure user, the Intimate user, the Escapist, the Narcissist, and the Discrepancy user based on the analysis of data from a sample of 51 participants, as explained below. This process could lead to a final version of such behavioural archetypes being developed based on qualitative analyses of the responses made by the participants, which were supported by quantitative analysis, to reflect the reality in mathematical terms and provide a detailed description of these archetypes. Furthermore, these behavioural archetypes can be enriched with information relevant to internal characteristics for each archetype, emotions, and psychological states, as discussed in phase three.

For the purpose of confirming that the behavioural archetypes were inferred from the analysis and whether there were seen helpful for people to recognise their problematic attachment style, we carried out a second diary study with 51 participants. In addition, this diary study was also performed to confirm whether the behavioural archetypes constitute a meaningful way of categorising social media users and can be used to generate new interventions and initiate discussions.

Convenience and snowball sampling techniques were used to recruit participants. We applied the same eligibility criteria and self-assessment as in the first phase when recruiting participants. About 80 people responded to our call for participants, through email or by telephone. We excluded 11 participants whose responses to the screening questionnaire suggested that their use of social media was not problematic. Eighteen of the remaining 69 participants dropped out during the study, leaving a final sample of 51 participants who completed the study procedure in full. 

The participants were given instructions on how to complete diary entries and a printed diary notebook. The diary notebook was designed to last for five days, and everyday participants were required to complete three entries a day and were identified as “morning”, ”afternoon”, and ”evening”. The recording times were selected in order to demarcate the reporting periods equally distributed over the course of the five days for the effective observation time series present in the analysis without overloading the participants with a more dense recording schedule, which may have been inconvenient. Participants were asked to spend two to three minutes completing each diary entry. Participants were instructed to leave an entry blank if they missed a recording time, rather than complete it by recalling.

Before the participants began to report, they were asked to spend some time reading the descriptions of the five archetypes and work out which archetype or archetypes fit them best. Having decided this, they were expected to follow the colour code for the entire reporting (there is a distinctive colour code for every archetype and the days were numbered from one to five). The five behavioural archetypes describe the predominant relationship styles that people display with social media. However, it is quite important to be vigilant on some participants who may find it difficult to match to one of these archetypes, since they are non-compatible with their corresponding online style. In such cases, it is important that they complete the template based on their interaction style. Participants were sent a daily reminder email to prompt them to record an entry in their notebook. To ensure confidentiality, each participant was assigned a unique code.

Upon the completion of the validating study, the participants were requested to provide clarification of some of their experiences with the five behavioural archetypes. We concluded that these archetypes are a useful way to categorize people with a problematic attachment to social media because all participants identified with at least one of the archetypes and two participants identified with two archetypes. In addition, participants’ feedback on the archetypes indicated that they found them helpful in categorising their behaviour and recognising problematic online behaviours. This feedback strongly suggests that the five behavioural archetypes are valid. We also used the data to validate the dimensions used for users segmentation. 

After confirming the five behavioural archetypes, we enriched them based on participants’ feedback. The Findings section summarises the behavioural archetypes and the main differences between the archetypes. The qualitative feedback in Section 5 summaries the feedback from participants on the five behavioural archetypes and Section 6 illustrates the quantitative validation data. 

## 4. Problematic Attachments to Social Media: Our Five Behavioural Archetypes 

Based on the four phases, we developed five behavioural archetypes for problematic attachment to social media. Each archetype contained the following information:

- Description: a brief overview of the behaviour archetype.
- Internal characteristics: attachment style, identity disclosure, social activeness, online behaviours, and personal attributes.
- Emotional states and examples: common user experiences and examples of associated emotions. Users’ experiences and associated emotions are split into positive and negative emotions. We utilised Parrott’s framework [77] to differentiate between primary, secondary, and tertiary emotions. The components of this section of the behavioural archetype were based on our previous research results in [73] on the relation between usage experiences and emotional states for people with problematic attachment to social media.
- Psychological states and examples: common user experiences and examples of associated psychological states. The components of this section of the behavioural archetype were based on our previous research results in [74] on the relation between usage experiences and psychological states for people with problematic attachment to social media.

Table 3 describes the behavioural archetypes that were developed for people with a problematic attachment to social media and Table 4 illustrates the key differences between archetypes.

The following subsections describe each behavioural archetype in more details.

### 4.1. Secure Behavioural Archetype

Users with a Secure behavioural archetype have several means of interacting on social media that allow them to feel safe and confident, which is a strong reason for their online attachment. This was found to be the most common behavioural archetype among our participants, who also had several online behaviours in common, as discussed in detail below.

- Identity: Their online identities usually reflect their real offline identity through the use of their real name and image. People of this archetype possess self-confidence. Revealing their true personality online is an effective means of ensuring their popularity and forming friendships. This may directly affect their behaviour on social media, including their attachment: “I suppose my online identity while I am active online on Facebook and WhatsApp is a secure identity, which reflects my real character and personality, as well helping to build trust in social media.” The core characteristics of this archetype are the ability to trust in others and positive expectations of both self and others: “I feel happy and secure when people are there when I need them”.
- Common features of this archetype include a desire to search for information through social media, due to its accessibility, which can promote joy, particularly if the information is positive. By contrast, a lack of information leads to feelings of fear and loss.
- This archetype is also socially active, sharing information online and forming friendships within closed groups. This is due to being deterministic, which increases their social capital and social ties [78], prompting feelings of security, which influence their use of social media as an essential aspect of their daily life. However, this may also result in feelings of isolation when faced with any decrease in interaction within these groups or when subjected to discrimination or exclusion from these groups. Exclusion from online social groups may result in negative emotions, including nervousness, anger, or a lack of happiness: “the Internet connection in my residence is so weak that I could not interact online with my friends, which made me feel isolated from the world”.
- There are a number of emotions and psychological states that are prominent in the previous points (i.e., the positive interaction with social media profiles through comments or positive feedback) which revolve around a sense of liking and satisfaction. These can improve people’s self-perception, leading to higher levels of self-esteem [79], and are thus capable of triggering the evolution of an online presence. Hence, easy online communication with relatives and friends (including sharing of news) has a positive impact on the emotions of Secure users: “I feel really happy because I had a great chat with my family and friends and found lots of things online that made me joyous”.

Despite these positive emotions, Secure individuals may feel regret or anger when they spend a great deal of time using social media as a result of their attachment. Anxiety arises when the content of social media is unpleasant, boring and repetitive, or results in negative thoughts due to the voyeuristic use of profile functions: “there was not anything else to see on Instagram or Snapchat; I felt bored”. Table 5 will present indepth details about Secure archetype. 

### 4.2. Intimate Behavioural Archetype

The Intimate archetype is composed of a specific attitude, often characterised as a commitment to concrete attachments and relationships and development of ethical strength through fulfilling commitments, even when doing so demands significant sacrifices and compromises, e.g., spending more time online and responding fast to maintain peer trust and relatedness. The patterns and behaviours of this archetype’s interactions with social media are as follows:

- The Intimate user’s online identity is real, and his or her real name and a genuine self-image are used in profiles, i.e., self-disclosure [80]. In addition, Intimate users frequently update their profile image because they want to show their friends and social media contacts their appearance. This helps them to make new friends, and they like to diversify their friends from different cultures and geographical areas, and this makes them feel happy and joyous: “I took some pictures of myself and decided to use a new one for my profile to share with my family and friends.”
- The core of the Intimate archetype is kindness in online interactions. The Intimate user feels confident and trustworthy and pays attention to others, helping them to address their difficulties: “I am always a good listener and my friends and relatives like talking to me about their feelings and problems”. Furthermore, the Intimate user’s emotions are explicit throughout his or her online interactions.
- Very active in terms of appearance and participation with others on social media. This helps Intimate users gain a reputation and form friendships, which may facilitate the evolution of problematic attachment. Friendships are satisfying, but comparisons with social media peers may provoke jealousy or envy: “I was jealous when I saw my friend’s posts, and I compare them with my day; I wish I could be like him”. Intimate users also experience anxiety and a loss of interest if faced with disagreeable friends or uncomfortable online content. This results in feelings of dislike and neglect: “I did a favour to someone who knows me from Facebook. Later on, he started sending lots of messages, which I disliked”.
- Intimate users feel positive and satisfied as a result of their online interactions. This leads to a secure attachment to social media, particularly in the context of friendships. It enhances the evolution of their online presence and makes them feel safe. Curiosity is also important and their attachment to social media may result in fear of missing out. Intimate users are eager to know what is taking place around them in the online world, especially their close circle.
- Intimate users may feel a kind of depression if one of their online friends is no longer available or when they engage in downward social comparisons with others; this leaves a feeling of lack of social support. They may enjoy new friendships yet also feel regret or anger about the amount of time they spend on social media: “I feel regret; I have lost precious time on social media” or consider that they spend too much time posting and disclosing details of their personal life. Table 6 will present indepth details about Intimate archetype.

### 4.3. Escapist Behavioural Archetype

The Escapist archetype represents a personality that is dependent on social media as a means of escaping real-life problems, anxiety, and expectations [81]. Social media then become an essential gateway to the online world. The Escapist uses social media as a temporary escape from real life problems and stress: “sometimes you experience difficult circumstances. Using social media and interacting with friends are crucial to getting through such periods”. Escapists’ use of social media has a number of common characteristics, including the following:

- The Escapist is often anonymous online, due to an unwillingness to form friendships that are more than temporary and because the practices of their real life are aimed at adjusting their mood and obtaining some entertainment. This gives the Escapist positive emotions, such as joy and pleasure, but they may feel regret if these positive emotions are delayed for a long period of time. The Escapist tends to be unconscious of their online interaction, leading one to ignore friends communications on social media or to procrastination and, hence, leading to negative emotions, such as sadness: “Sometimes I use social media unconsciously, by which I mean that I see some friends texting, but I am in the kind of mood where I’ll open and read their messages but not respond and then feel sad”.
- The Escapist may also have some kind of self-discrepancy [82]. The Escapist’s online personality may differ considerably from their real personality; they may pretend to be happier or younger online: “Online interaction sometimes forces me to respond to people I do not want to talk to”. Escapists may play a role and act in order to garner sympathy and boost their self-confidence.
- Escapists have an avoidant attachment to social media. They wish to be self-reliant and, therefore, do not aspire to form deep friendships with others through social media, choosing instead to entertain themselves in their own way [83].
- Some of their patterns of use may cause side effects resulting in negative emotions. For example, Escapists may categorise themselves according to factors such as interests, age, gender, or membership of an occupational group: “I am trying to contact journalists who with a similar background”. This approach may lead to loneliness and isolation, accompanied by other negative emotions, such as sadness, anger, and regret, with significant negative consequences for their self-conception. Table 7 will present indepth details about Escapist archetype.

### 4.4. Narcissist Behavioural Archetype

The Narcissist archetype often wishes to share their successes, dreams, ambitions, and achievements with their social media friends, firstly, because they believe that their friends are eager to hear about their activities and secondly, to elicit positive emotions and comments [84]. This personality is closely tied to social media. Narcissists feel safe when they are online and are made anxious by any loss of online activity. The Narcissist archetype is associated with positive self-views and self-concepts, resulting in a relatively high number of friendships, fairly heavy self-promotion and self-presentation. The characteristics of social media-related behaviours of Narcissists include the following:

- Narcissists struggle to resist using social media and respond promptly to messages, taking part in conversations, comments, or the exchange of information. They tend to manipulate and update their profile content, including posting and changing their profile status: “I shared something via Facebook and I saw that everybody liked it; that made me happy”; “As soon as I woke up I used my social network and shared a lot of pictures and videos. I felt amazing today”. This interaction pattern may result in an evolution of their online presence and anxiety may arise from concerns about the expectations of others, perhaps leading to negative emotions such as nervousness, worry, and shame.
- Narcissists use their real identity for their online profile. This is a self-presentational choice and brings joy and satisfaction. Narcissists use a genuine self-image on their online profile, because they typically believe themselves to be attractive, both as appearance and lifestyle, and so they think that this will help them to be noticed and so achieve their social identity goals: “I have changed the profile picture I use on Facebook for family, relatives, and friends, because I took a new picture of myself”.
- Narcissists are influenced by their peers, leading to a form of competition in the use of social media, i.e., they are easily manipulated and feel they are at the centre of any interaction. Their self-categorisation is predictable, which results in them comparing themselves with others and may lead to negative emotions, such as envy and jealousy. Table 8 will present indepth details about Narcissist archetype.

### 4.5. Discrepancy Behavioural Archetype

This is one of the minority archetypes discovered in our study. It represents individuals who are characterised by a sense of being different from others in terms of social media habits. There are a number of notable patterns in Discrepancy users’ social media interactions:

- Discrepancy users’ profiles are frequently linked to their real identity because they see themselves as different from others and tend to classify themselves according to their feelings of self-worth and their self-perception: “I am trying to be myself. Also, I like to share posts with my friends who have the same interests”. Their attachment to social media takes an avoidant style, due to their focus on creating and maintaining self-esteem, in a number of different ways and on different levels, using the features and functions of social media. They may experience regret or anger as a result of the amount of time they spend online, on such activities.
- They also feel disturbed and lose concentration when their thinking is dominated by online activities to the extent that they are unable to focus on anything else. This leads them to lose interest in using social media, giving rise to negative emotions, such as nervousness and anger: “When I was checking my message I lost my concentration, and I missed an important task, which makes me feel anger”.
- Their interactions are unlike those of other behavioural archetypes, in that they are discriminating and selective when it comes to content posted to their accounts (including posts or comments) because they suffer from social anxiety: “I care about my appearance. I want people to see me as good looking and happy, even if that is far from the reality”. In addition, they expect a lot from their online friends, which makes them feel anxious and lonely and may lead to other negative emotions, such as fear and nervousness: “I was expecting a message, and I felt lonely while I was waiting”. Table 9 will present indepth details about Discrepancy archetype.

## 5. Evaluation of the Behavioural Archetypes: Analyzing Participants Feedback’s

As previously stated, the diary study design covered how people aligned and reacted to the five behavioural archetypes and, additionally, how these behavioural archetypes influence users with regard to their problematic online attachment. In this section, several points were extracted through the analysis of the interviews we conducted with 18 participants who completed the second diary studies. The participants were chosen to cover the different behavioural archetypes and also consider the qualitative comments they gave when they completed the diary notebook. The interview asked the main question around how they found the description of the archetype and whether they have any suggestion to improve it. Their views are summarised in the following points:

- Representative nature: Participants believed that these behavioural archetypes were able to capture the main problematic online attachment style, making it easy for them to liaise to one of these archetypes. In addition, they began to understand and predict patterns of interaction with people around them. This can be attributed to the nature of the building components of the developed archetypes, which makes them closer to reflecting reality. Examples of the comments received included “I definitely can find one more characteristic that I have it myself in behavioural archetype number four” and ““I found that easy to put myself in one of these behavioural archetypes, which is good to see my usage in front of me”, and “The five behavioural archetypes are well-structured and put together. I found it relatively easy to link to one of them and populate my daily diary”. The representation nature of our five archetypes is also supported by the fact that each of the recruited participants, the 51 participants of the second diary study, found themselves in one of the archetypes.
- Raising awareness towards the interaction style: Participants agreed that the diary study and their completion of the sheets on a daily basis made them more conscious about their online behaviours and their problematic online attachment. They started to think of managing it during the diary study period. Examples of the comments received on this aspect include “To be honest, these behavioural archetypes prompted me to think about how to use social networking sites” and “I would like to thank you because I started thinking about social media effects”, and “I realised my patterns are from behavioural archetype one, which exactly reflects my interaction.”
- Terminology awareness: Some participants thought that there was an overlap between emotions and psychological states in each of the five behavioural archetypes, some of which are difficult to distinguish and needed further clarification. For example, participants mentioned they tended to see negative emotions and some psychological states as a similar thing, occasionally. They also found the subtle differences between emotions hard to recognise, and some could not differentiate between certain emotions of a similar nature, such as anxiety and regret and sadness. This suggests that an induction around emotions and the psychological states would be needed when behavioural archetypes are used for user modelling and behaviour awareness.
- Engaging presentation: At the start of the diary study (phase four), participants were presented with a summary description of each of the personas written in a simple and concise format. They were asked to liaise themselves with one or more of the behavioural archetypes. Based on their choice, participants were then given a diary book including entries tailored to the detailed description of that archetype. While participants found the detailed form helpful to self-diagnose their actual experience, they also expressed that the questions were of a “dry nature” and “heavy at times”. Hence, a more user-friendly and lively format of the behavioural archetypes would need to be presented to people if the intention is to use the behavioural archetypes for design or diagnosis purposes, to avoid causing a tiring and less engaging experience.
- Behavioural archetypes’ live presentation and representation: A set of live behavioural archetypes were created in response to the previous point around the need for an engaging presentation. The behavioural archetypes were presented in the follow-up interviews. However, some participants expressed concerns about the names of the behavioural archetypes, especially the Narcissist and the Paranoid. We stress here that these names were hidden from the actual studies and only used at that phase for consultation. Still, we replaced Paranoid with Discrepancy. Participants also felt that demographic data added to each behavioural archetype could be seen as stereotyping. The archetype names used in this paper are meant for the practitioners and researchers, and we would need more user-friendly terminology and presentation if used for other purposes, such as validating a design or eliciting user requirements. In addition, it appeared that assigning gender and age to a behavioural archetype may deter some from choosing it or liaising themselves naturally to it.
- Objectivity and influence: Participants noted that they could be biased in filling the diary and recognising their behavioural archetypes altogether. For example, one participant mentioned that emotion could be volatile depending on the different interactions they have on their different social media accounts, and, at times, they may feel “different emotions simultaneously”, according to the various messages and content received. They also noted that “being in a negative mood due to a real-world event can expand to the low mood in using social media and vice versa”. Hence, the emotions and psychological states associated with the use of social media should not be attributed to that use entirely. A more objective measure of that relation, other than the self-report, is, therefore, needed.

## 6. Quantitative Validation on Behavioural Archetypes

The diary entries used to validate the behavioural archetypes were transferred to numerical form for statistical analysis (We performed statistical analysis to help validate the five behavioural archetypes and to make the definitions of the behavioural archetypes more rigorous and scientific. As a theoretical framing for the evaluation, we use the Goal Question Metrics (GQM) proposed in [85]. Table 10 explains GQM instantiation for our quantitative validation. Here, we analyse the diary entries for the 51 participants of Phase four of our study, as this was a study that we conducted to validate the archetypes.

### 6.1. Sample Overview

As part of the earlier qualitative phase of research, all participants were asked to select one of five behavioural archetypes that best reflected them. Each participant was subsequently asked to undertake a survey three times a day (morning, afternoon, and evening) for five days, providing a more ecologically valid sample comprising 15 separate responses for each question on the survey. All questions were binary and the process of data transfer is summarised below in Table 11.

Questions were designed to elicit attributes according to the self-assigned behavioural archetype, and, hence, each different archetype had a different number of questions. All questions within each Behavioural Archetype were based on themes highlighted by preliminary research [73,74]. The differing number of questions related to how relevant each theme was within each behavioural archetype. Specifically, these results are shown below in Table 12.

Question responses were aggregated to create four independent variables, each of which corresponded to a section of the survey. The first five questions on each survey measure the internal characteristics of the archetype chosen by the participant. It is from these responses that the internal validity independent variable was derived. Similarly, the questions on the survey corresponded to the positive emotions, negative emotions, and psychological states from the dependent variables. 

The raw data comprised 51 participants who undertook the diary response survey. As part of the assessment of the data, a “count” variable was created to identify the number of non-responses. Six participants were removed as outliers—four for insufficient data completion and two for zero-response variation. Thus, the final data set taken forward comprised of 45 participants. Table 13 and Table 14 below illustrate the split for each of the three independent variables; behavioural archetype, Gender, and Age group.

### 6.2. Quantitative Validation of the Behavioural Archetype

It seems reasonable to consider that if the Behavioural Archetypes are not a valid concept, then it follows that each of the five Behavioural Archetypes would be equally likely to be chosen when selected by participants—in effect, selecting a Behavioural Archetype would be purely down to chance.

The Chi-Square test provides a way of assessing the “goodness of fit” for our sample under a theoretical distribution, where each behavioural archetype had an equal chance of being selected. Table 15 will present Chi-Square value for equal proportions of participants.

As seen from the table above, there was good evidence (*p* = 0.018) against the null hypothesis that Behavioural Archetypes were equally likely to be chosen. We, therefore, accept the alternative hypothesis that all Behavioural Archetypes are not selected with equal probability. This quantitatively validates a perceived difference in Behavioural Archetypes on behalf of the participants. 

A Chi-Square test was also used to assess the gender balance of the study. This is pertinent to the desire for the Behavioural Archetype concept to be adaptable and applicable to a large population. Any significant findings could be limited if the sample is not gender balanced, so it is important to establish whether the sample was prone to gender bias in participation. There was no evidence to reject the null hypothesis (*p* = 0.423) that the probability of the gender of a participant is equally likely. We, therefore, observe that the overall study is gender-balanced.

### 6.3. Descriptive Statistics for Each Behaviour Archetype

Having verified the entity of the Behaviour Archetype, a quantitative analysis of the composite features for each behavioural archetype was undertaken. Necessarily, the starting point for this analysis is the descriptive statistics for each behavioural archetype. Due to the varying number of questions on each dependent variable, a statistic was created for each individual, representing the proportion of responses that were felt—essentially a mean response value. These were subsequently used as the basis for the descriptive statistics outlined in Table 16. The proportion of felt responses for each individual were represented by dependent variables: The Internal characteristics variable (PIVPERC), Positive emotions variable (PPOSPERC), Negative emotions variable (PNEGPERC), and Psychological states variable (PPSYPERC).

The features of each behavioural archetype were analysed using these dependent variables as the basis for statistical inference.

### 6.4. Internal Characteristics Validity Measure for Behavioural Archetype

The first five questions of each archetype were questions that assessed the extent to which the archetype chosen by the participant, in fact, reflected that behavioural archetype. These form the basis for testing the internal validity of each archetype. The Tables below show the mean proportional response according to each of the independent variables (Gender, Age group, and Behavioural archetype).

It would appear that gender had no impact, as the means are broadly the same, as shown in Table 17. There is a noticeable increase in internal validity as the age groups increase from 0.56 to 0.65 and 0.71 through increasing age groups (refer to Table 18 for more details). Each archetype was broadly similar, with means ranging from 0.58 to 0.68, as in Table 19. The Discrepancy mean value for internal validity is notably lower than the others, although this is not a test of significance.

### 6.5. Stability of Behavioural Archetype

**Figure 3:**
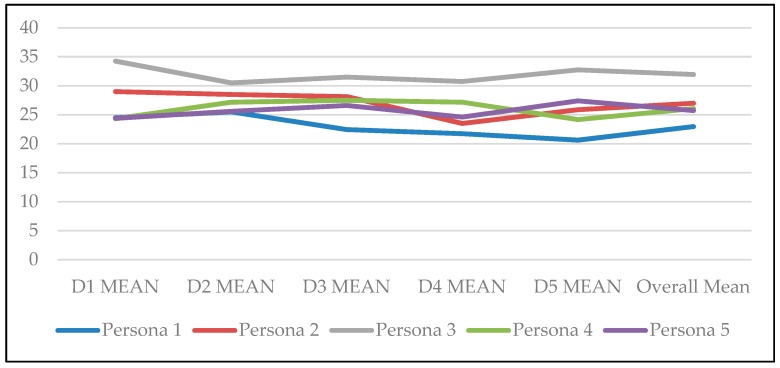
Temporal stability of behavioural archetypes.

An additional requirement for each behavioural archetype to be valid is that it must be stable. Table 20 shows the data for the daily mean of all diary questions felt for each behavioural archetype. These have been plotted on a line graph (as presented in Figure 3) and appear to be fairly stable. 

## 7. Discussion

This four-phase study has identified five different behavioural archetypes describing the distinctive patterns of problematic attachment to social media: Secure, Intimate, Escapist, Narcissist, and Discrepancy. Each archetype has specific characteristics and behaviours, each linked with emotions and psychological states. A common understanding is that behavioural archetypes, when used for designing the user experience, have the following advantages [86,87]:

- They facilitate the design process that engineers connect to a human face and name rather than the abstract user data.
- They provide a common, fast, and effective form of communication between software engineers and designers.
- They help engineers maintain a focus on a limited subset of users (archetype) at a time that can result in more robust design decisions.
- They are useful for system validation purposes, where proposed designs, features, and solutions can be examined against the needs defined in the archetype.

In light of the advantages referred to above, behavioural archetypes can be adopted as a design tool to guide the design process of online behavioural change tools, especially when such techniques have not received much attention from researchers and practitioners interested in Internet addiction, in comparison with the health and education domains [88,89,90]. As mentioned earlier, interventions need to be tailored to tackle the problematic attachment to social media in many aspects. This suggests that a Human Centered Design (HCD) approach should be used to understand individual needs and design solutions for them. As the archetypes were built with the internal characteristics of the users, their emotions, and the psychological states accompanying their social media experiences, all these factors present a high degree of similarity to the common grounds used to create goal-directed and engaging personas. In other words, the archetypes can be easily translated and mapped into these types of personas for the design team to guide the ideation sessions for the intervention design.

There are other expected uses of our proposed behavioural archetypes. For example, the intervention designers and researchers can use these empirically based archetypes as baselines to quickly establish an image of the targeted audience and develop potential scenarios before identifying and engaging any end-user showing problematic attachment [38]. Once the representative users are identified, they can also be used to elicit the users’ preferences and design elements towards problematic online attachment, as well as raise awareness of their usage style [91]. Moreover, they can be used in conjunction with different intervention techniques and models (e.g., GROW: Goal; Reality; Options; Way forward [92]) to design archetype-based interventions. Users would be able to set their goal and specify their situation using one of the five behavioural archetypes, and this information would be used to identify tailored behavioural recommendations, options, persuasive techniques, and coping strategies that could be used alongside relapse prevention strategies to help users manage their use of social media and improve their wellbeing. 

Our archetypes can serve as reference user models for discussion around the concept of problematic usage of social media. Internet addiction is still not classified as a mental disorder, and evidence around internet gaming disorder is still required to be included formally in the Diagnostic and Statistical Manual of Mental Disorders (DSM) [93]. This indicates that further research in this area is needed in order to gather evidence that can be used to compile clinical psychological diagnostic criteria to confirm technology addiction, whether for games or social networks [20]. Our work is the first attempt to establish empirical evidence for social network addiction, which can be later used to create relevant diagnostic criteria.

Our study indicated that people experience various psychological states, including anxiety, depression, and a lower sense well-being in combination with their social media usage. We obtained such evidence through the usage of diary studies, which mainly helped us capture the lived experience and ecological validity of the data collected. Through the studies presented in this paper, it was acknowledged that social media is used as a vehicle to provide social support, return a sense of connectedness among users, and promote social capital and self-esteem. However, it was noted by the participants belonging to all of the archetypes that some design features seem to rely on gratification through positive expectations, social recognition, feedback, and rewards to persuade online interaction and increase usage. While we are not advocating a causal relation between social media usage and negative psychological and emotional states, one of the possible triggers can relate to the design features.

Despite the positive outcomes of online interaction through social media, social media may also be linked to negative impacts on well-being [94]. Ultimately, a design that promotes positive wellbeing can support users’ values, e.g., social satisfaction and system trustworthiness and trust. The design could play an essential role in promoting and helping healthy online interaction. For example, current social media design allows users to accept or reject friend requests and set up privacy settings that enhance trust in the system. The design of wellbeing tools will be fundamentally different according to the user model. For example, it is apparent that the main reason for the Secure archetype to be overly attached to social media is to stay in control by remaining connected and attentive, and, hence, tools like recap and filtering would benefit them. People belonging to the Intimacy archetype are more susceptible to being emotionally hurt if communications and social interactions are not up to their expectations. Hence, tools of consolation and expectation management would help to increase their digital resilience.

Users with problematic attachment are characterised by a denial of their online behaviour, reactance when they feel their freedom to continue using social media is controlled by some measures like reminders and timers, and relapses when they try to regulate that relation both in terms of usage style and the preoccupation around it [41,42,43]. Thus, the design of social media needs to consider the differences that users may have in their characteristics based on different configurations of software-based intervention, to facilitate the social welfare of users [95]. Our future research will combine personality traits of users and their behavioural archetypes in order to customise intervention and help decision makers, e.g., mentors and the users themselves, through recommendations and default options.

In addition, users showing problematic attachment to social media may experience a craving to increase their reputation or feel anxiety when unable to connect or interact as they wish; this desire relates to the Narcissist and Intimacy archetypes. We found a set of negative psychological states that are facilitated by features of social media; these archetypes include grouping, conversation, relationship, and reputation. This suggests that the current design adopted by social media may impact negatively on users’ wellbeing with the absence of any countermeasures to help mechanisms like self-regulation and mindfulness. Suggestions have been made that individuals with poorer wellbeing are more likely to use social media in order to alleviate loneliness. However, a recent large-scale longitudinal study found that greater use of Facebook was associated with poorer future wellbeing and that this correlation was stronger than the one between real-world social interaction and positive well-being [96].

Regulating ones relation with social media can conflict with the innate desire to use them. For example, people from the Escapism archetype constantly expressed the need to modify mood and avoid reality by creating an alternate reality on social media. Hence, the design of tools to help people in that regulation should consider the conflicted requirements of people typically found when trying to regulate addictive behaviours. Participatory design has been used successfully in the development of interventions, such as in improving the dynamics and challenges of serious games for health promotion [97]. Participatory design methods [98] may, therefore, provide a solution to reducing this conflict in order to develop platforms that are acceptable to both users on the one hand (in terms of ease of use and satisfying needs) and developers on the other hand (in terms of promoting wellbeing, in line with corporate social responsibility).

However, with participatory design methods, there are a number of issues to be taken into consideration. Care needs to be taken when including individuals with problematic behaviour in the design of interventions, to ensure that such interventions provide an appropriate message for the target population (See Section 5 for some of the issues). For example, such individuals may be in denial regarding the extent of their issues [99]. They may, therefore, trivialise the seriousness of the problem, suggesting that unusually high levels of social media usage are normal. Despite these issues, co-design has been successfully implemented with individuals with problematic behaviour. For example, in a recent study, a workbook to address alcohol use when quitting smoking was successfully created with input from individuals who drink alcohol to hazardous levels [100]. This design method, therefore, has the potential to work with individuals who have a problematic attachment to social media. Our archetypes are intended to help in that process. Indeed, as highlighted in Section 5, participants realised their problematic attachment styles more when they participated in our diary study and had to fill in the entries for their chosen archetype.

### Limitations

The research utilised convenience sampling and all participants were volunteers, which may have biased the sample. It would also provide more validity if the sample size was higher. However, we stress here that the initial version of the archetypes was, itself, based on a substantial qualitative study. In addition, the set is supported by previous studies, so their creation was both empirical and literature-based. The results of our study are not meant to be a generalisation of the typical patterns of problematic attachment to social media. Instead, we advocate their usage as a starting point for a variety of processes, such as the requirements of elicitation, personalisation, tailoring, and self-diagnosis, both for the behavioural change processes and the tools supporting these processes. Our participants self-declared to have a problematic attachment to social media. This is another bias in the sample. Having people who may exhibit symptoms of problematic attachment but are in denial of it may have revealed other kinds of attachment and, hence, archetypes. However, we would need a different research method design to deal with such a user group, as a diary study is based on self-reporting and user cooperation. Our design of the diary study collected data three times a day and used reminders. While this process helps to minimise recall bias and increase ecological validity, there could be still a degree of imprecision, especially because some may encounter conflicting feelings during the same period of the day. To solve the last two limitations, we will be looking at more objective ways to collect user experiences in the future, such as using multimodal interaction and affective computing techniques.

## 8. Conclusions

In this article, we reported a four-phase study for understanding the problematic attachment to social media by capturing users’ as-is experiences based on diary studies. We also developed a set of five behavioural archetypes to represent users with problematic attachment to social media. These archetypes are: Secure, Intimate, Escapist, Narcissist, and Discrepancy. To our knowledge, this set of archetypes is the first empirically based way of clustering users with problematic attachment to social media. These archetypes can be used to not only better understand and segment users with problematic attachment to social media but can also be used as a tool for facilitating effective communication between different teams and designing tailored interventions to address problematic attachment to social media in a Human Centered Design (HCD) approach. Our next step is to explore how the developed archetypes can be used in a design process to facilitate behavioural change through (1) developing a tailor intervention for problematic attachment to social media and (2) supporting existing therapies, such as motivational interviews [101] and Goal Setting [102], so that they are used as a baseline for the discussion. For (1), we will pay particular attention to investigating the effectiveness of using our behavioural archetypes as a design tool for creating behavioural change software for people with a problematic attachment to social media. A method will be devised accordingly to enable the design team to use our archetypes to develop tailored and personalised software. This includes, but is not limited to, using the archetypes as the foundation to create personas and scenarios. For (2), we will investigate how our behavioural archetypes can benefit the behavioural change process in terms of intervention planning and assessment. For example, a question would be asking whether choosing a subject from one of the archetypes to represent them would help the subject and their mentor in understanding the issue and setting up a plan accordingly. Our ultimate goal is to improve the archetypes through its usage to help us get more insight and propose solutions around the utilisation of the behavioural archetypes as a tool for people with a problematic attachment to social media, in order to raise their awareness and help them manage their attachment.

## Figures and Tables

**Table 1:** The segmentation process for users with problematic online attachment.

Online Behaviours	Online Attachment Style		
	Secure	Fear of Missing Out (FoMO)	Avoidant
Self-enhancement			Segment 3
Irresistible urge and self-disclosure		Segment 4	
Categories themselves			Segment 5
Tracking information	Segment 1		
Kindness and self-presentation		Segment 2	

**Table 2:** Five users’ behavioural archetypes: initial segmentation.

Online Behaviours	Behavioural Archetype for Each Segment		
Self-enhancement			Escapism archetype
Irresistible urge and self-disclosure		Narcissism archetype	
Categories themselves			Discrepancy archetype
Tracking information	Secure archetype		
Kindness and self-presentation		Intimacy archetype	

**Table 3:** Descriptions of the behavioural archetypes.

No. #	Archetype Name	Description
1	Secure archetype	This archetype likes to feel assured. Social media helps them to maintain this feeling by building successful relationships that increase their connectedness and presence. Despite this, they can occasionally lose their sense of security, i.e., when unable to access social media, interact with peers, or express themselves and receive the responses they feel they need to maintain their desired level of social presence and connectedness.
2	Intimate archetype	These individuals become closely attached to their social media and their online friends. This commitment to their friends manifests as a keen interest in their activities and (when appropriate) empathetic responses. They know what they like and refuse to engage with the material of which they disapprove. Their natural curiosity and vulnerability to the fear of missing out can lead them to become anxious if they are unable to maintain an online presence.
3	Escapist archetype	These users employ social media to avoid the reality of their own lives. They may engage in anonymously or create fictitious online personalities. They have little desire to form real relationships offline and use social media for entertainment. Their behaviour could easily exacerbate their true loneliness.
4	Narcissist archetype	These individuals pay excessive attention to the thoughts and opinions of others about them on social media and they often seek approval from others. They lack the confidence to use their real identity on social media but have an urge to respond as soon as possible to updates. Social media are used as a way of competing with others, but there is potential for discontent or jealousy if they feel their contacts are experiencing more enjoyment or achieving more than they are.
5	Discrepancy archetype	This archetype spends a considerable amount of time attempting to boost his or her self-esteem on social media, but this frequently leads to feelings of regret, including the feeling of having wasted time that could have been spent doing other things. Even when engaged in other offline activities, these users are likely to think about social media, which can have an adverse influence on their daily lives. In addition, they may feel excluded and alone if social media contacts do not live up to their high expectations.

**Table 4:** Key internal characteristic differences for the five behavioural archetypes.

	Internal Characteristics	SM Boosts the Feeling of Being Secure	SM Boosts Perceived Security and Fear of Missing out	Avoidant Online Attachment Style	Committed to Online Group	Socially Active	Tracking Information	Self-Presentation	Kindness Role	Trust and Positive Expectation	Self-Enhancement	Procrastination	Self-Discrepancy	Vulnerable to Peer Pressure	Irresistible Urge	Disturbance and Loss of Concentration	Categories Themselves	Detached from Reality
Online Behavioural Archetypes																		
Secure archetype		◯			✕	✕	✕											
Intimate archetype			✕					◯	✕	✕								
Escapist archetype				◯							✕	✕	✕					
Narcissist archetype		◯						◯						✕	✕			
Discrepancy archetype				◯												✕	✕	✕

**Table 5:** Overview of the Secure behavioural archetype.

**Secure Behavioural Archetype**		
**Internal Characteristics**	**Description**	
Identity	Feels confident using information that identifies him/her in interactions online, e.g., real (or close to real) name, real (or close to real) picture, along with location, place of work, and email address	
Social media boosts the feeling of being secure	Interacting online contributes to a feeling of safety and confidence. Value peer support and continual presence	
Tracking information	Considers it important and worthwhile to search for events, feed requests, and news on social media. Keeps up to date with information and has a reasonable response time	
Socially active	Is active on social media (i.e., posting and commenting) and enjoys being involved in groups and establishing new connections	
Committed to their online group	Likes to maintain relationships with others and tolerates situations in which this may require acceptance of different attitudes and styles of interactions	
**Examples of Usage and Associated Emotions (Positive and Negative)**		**Emotion Example**
Positive usage experience	Social media used as a medium for reciprocal messaging, posting and commenting, i.e., interactive social communication	Satisfaction, liking, joy
	Social media as an accessible facilitator of activities related to pleasure and entertainment	Joy
	Social media help communicate with relatives and friends, as well as sharing information and contributes to a sustainable sense of connectedness and presence	Happiness, joy, astonishment
Negative usage experiences	Using social media for longer than required	Regret, anger
	Limited or no access to social media due to connectivity problems or restrictions imposed by the social context	Nervous, anger, fury, unhappiness
	Not receiving sufficient or timely responses from peers when looking for support or socialisation	Anger, sadness
	Fear of missing out on certain events, news, opportunities, and timely interactions	Worry, fear, jitteriness
**Psychological States**	**Usage Experiences**	
Loss of interest	When information content, interactions, and contacts do not change and when they become repetitive	
Anxiety	When spending too much time on social media or when dissatisfied with the content, interaction and unable to do much to change it	
Boredom	When there is nothing new on one’s social media which make them scrolling through content without conscious	
Loneliness	Being unable to connect and interact or receive responses as desired	
Craving	When there is a pressing need to shape and maintain one’s online identity and self-concept which in turn increases their reputation	

**Table 6:** Overview of the Intimate behavioural archetype.

**Intimate Behavioural Archetype**		
**Internal Characteristics**	**Description**	
Identity	Feels confident and needs to use their real name and image for online profiles; updates his or her profile picture regularly	
Kindness role	Has inner confidence and is eager to help others by listening to their problems and offering help	
social media boosts the feeling of being secure and FoMO	Comfortable when engaging online, but natural curiosity means that any interruption to online activities can result in fear of missing out	
Self-presentation	Confident about personal appearance and regularly changes his or her profile picture as a reminder of his/her presence and current status. This may also result in a tendency to compare their life with the perceived lives of others	
Trust and positive expectation	Believes that his or her online friends can be relied on and is, therefore, keen to interact with them	
**Examples of Usage and Associated Emotions (Positive and Negative)**		**Emotion Example**
Positive usage experiences	Social media are a tool for communicating with friends and family	Happiness, joy, astonishment
	Social media are useful for reciprocal messaging, posting, and commenting, i.e., interactive social communication	Satisfaction, liking, joy
	Social media as a source of fun and entertainment	Pleasure, joy
	The Intimate user is well-liked and social media are a useful way of making many friends from various locations and backgrounds	Happiness, enjoyment, satisfaction
Negative usage experiences	Using social media for longer than required	Regret, anger
	Unwelcome communications received online, including disagreeable messages from friends, inappropriate subject matter, or comments with which one disagrees	Dislike, neglect
	Use of social media to compare one’s own life with those of one’s contacts	Jealousy, unhappiness
	Limited or no access to social media due to connectivity problems or restrictions imposed by a specific social context	Nervousness, anger, fury, unhappiness
	A continual need to know about contacts’ activities, resulting in fear of missing out	Worry, fear, jitteriness
**Psychological States**	**Usage Experiences**	
Loss of interest	This occurs when there is little change of content, interaction, and contacts, resulting in the experience becoming repetitive	
Boredom	Arises when they lose popularity due to an inactive profile and the same content being posted	
Anxiety	This could result from spending too much time on social media, being unable to access social media. The belief that contacts expect a quick response to posts or online activity may also provoke anxiety	
Loneliness	Arises if, over time, the Intimate user comes to rely on social media in daily life and an inability to communicate leaves him or her feeling excluded	
Craving	This can arise in the face of routine. They are using social media on a daily basis which then turns into a daily habit	
Depression	This can occur when they engage in downward social comparison with others in order to meet self-evaluation needs	

**Table 7:** Overview of the Escapist behavioural archetype.

**Escapist Behavioural Archetype**		
**Internal Characteristics**	**Description**	
Identity	Escapists prefer to remain anonymous during online interactions	
Procrastination	They may postpone responses to their online friends and, unconsciously, leave their messages and interactions unanswered	
Self-enhancement motive	Social media offer an escape from real life, allowing the Escapist to create an imaginary persona that boosts his or her self-image and allows him or her to be viewed positively	
Self-discrepancy	Escapists’ unhappiness with their real-life situation causes them to make false claims about themselves when online, for example giving a false age or pretending to be happier than they really are	
Avoidant online attachment style	Escapists are unwilling to form close friendships online, which may reflect an underlying lack of trust in those with whom they engage	
**Examples of Usage and Associated Emotions (Positive and Negative)**		**Emotion Example**
Positive usage experiences	Social media are helpful for communicating with relatives, friends, and sharing information	Happiness, joy, astonishment
	Social media are used for activities resulting in pleasure and entertainment	Joy
Negative usage experiences	Fear of missing out on certain events, news, opportunities, and timely interactions	Worry, fear, jitteriness
	Unconsciously spending longer online than one intended; avoiding communicating with others	Regret, anger, sadness
	Limited or no access to social media due to connectivity problems or restrictions imposed by the social context	Nervous, anger, fury, unhappiness
	Content or interactions do not suit one’s mood	Anger
**Psychological States**	**Usage Experiences**	
Anxiety	Occurs due to excessive usage of social media or due to displeasing content	
Boredom	Arises when the individual may engage in passive interaction such as viewing and scrolling in an unconscious mood	
Loss of interest	This arises when the same content is repeatedly posted on social media	
Loneliness	Arises if social media are crucial to social interaction and one is unable to engage and feels excluded	

**Table 8:** Overview of the Narcissist behavioural archetype.

**Narcissist Behavioural Archetype**		
**Internal Characteristics**	**Description**	
Identity	Narcissists are sufficiently confident in their online interactions to use one or more items of information that identify them, e.g., real name, picture, location data, workplace, and email	
Self-presentation	Narcissists have a high opinion of themselves and use social media to show off their good qualities, including their physical appearance, personality and achievements, e.g., they frequently update their profile content in order to attract the attention of others. However, this leaves them vulnerable to comparisons with others that they may find it difficult to avoid	
Peer pressure	They are keen to impress their contacts and so may experience competitive pressures. They desire to be the centre of attention, and remaining thus requires considerable effort.	
Irresistible urge	They have an urge to respond to new posts and conversations and exchange information and content as soon as possible	
Secure and fear of missing out online attachment	Engaging with others via social media makes them feel secure and confident. If they cannot access social media, they may become uneasy and experience fear of missing out	
**Examples of Usage and Associated Emotions (Positive and Negative)**		**Emotion Example**
Positive usage experiences	Social media are helpful for communicating with relatives and friends, sharing information and experiencing a sense of ongoing connection	Happiness, joy, astonishment
	Social media are used for reciprocal messaging, posting and commenting, i.e., interactive social communication	Satisfaction, liking, joy
	Narcissists are well-liked and social media help them to make many friends from many different countries and cultures	Enjoyment, satisfaction
Negative usage experiences	Social media-motivated comparisons between one’s own life and the lives of contacts, often in terms of their activities	Jealousy, unhappiness
	Curiosity about their contacts’ activities can lead to fear of missing out if one is unable to access social media	Worry, fear, jitteriness
	Limited or no access to social media due to connectivity problems or restrictions imposed by the social context	Nervous, anger, fury, unhappiness
	Undesirable social media content and comments are posted by contacts they consider disagreeable	Dislike, neglect
**Psychological States**	**Usage Experiences**	
Boredom	Arises when there is no new social media content or content is repetitive	
Loss of interest	Arises when social media contacts have failed to add any new content, or when one lacks time to access social media	
Loneliness	Social media are a key part of one’s social life, and one’s group memberships reflect one’s personal preferences	
Anxiety	Evoked by difficulty in accessing one’s profile or being unhappy with social media content. Anxiety may also result from a feeling of commitment to be a highly responsive and unconscious quick response	
Craving	In the form of a pressing need to shape and maintain one’s online identity, self-concept, and reputation	

**Table 9:** Overview of the Discrepancy behavioural archetype.

**Discrepancy Behavioural Archetype**		
**Internal Characteristics**	**Description**	
Identity	Discrepancy users use one or more items of information that identify them, e.g., real name, picture, location data, work place, and email, in online interactions	
Avoidant online attachment style	Discrepancy users are unwilling to form close bonds with people they engage with on social media and find it difficult to trust those they meet online	
Categorise themselves	Discrepancy users believe that they are special and contrast their own situation with their contacts’ situations by comparing profiles and activities	
Disturbance and lost concentration	The Discrepancy user finds that handling numerous interactions online simultaneously leads to a loss of concentration and so prefers to focus one interaction at a time	
Different from reality	The Discrepancy user behaves very differently online and in the real world	
**Examples of Usage and Associated Emotions (Positive and Negative)**		**Emotion Example**
Positive usage experiences	Social media are an accessible facilitator of pleasure and entertainment activities	Joy
	Social media are helpful for communicating with relatives and friends, including sharing information, resulting in a sustained feeling of connectedness and presence	Happiness, joy
Negative usage experiences	Frequent online engagement, accompanied by a lack of self-awareness and concentration	Regret, anger
	The fear of missing out on certain events, news, opportunities or interactions	Worry, fear, nervousness
	Failing to receive sufficient or timely responses from peers	Sadness
**Psychological States**	**Usage Experiences**	
Boredom	Arises when their interaction is passive and unconscious	
Anxiety	Provoked by spending longer than intended on social media or being unable to check one’s profile	
Loss of interest	Caused by the disapproval of others’ content and interactions, or because the content remains unchanged or becomes repetitive	
Loneliness	They categorise themselves, which can result in feelings of isolation, particularly if contacts have not been active online	

**Table 10:** Evaluating our behavioural archetypes through Goal Question Metrics (GQM) model.

**Goal 1** Are the diary study data valid at the unit level? (for each participant)	
**Questions** Has each participant responded fully to all survey questions over the five days period? Are questions responded to in an honest manner? Has a participant exhibited variation in their responses? Is that variation reasonable? Are there any patterns within the data that should not be expected? Can any visible patterns be rationalised? Alternatively, should the participant be considered an outlier and their data removed?	**Metrics** Response Rate was calculated for each participant to identify potential outliers. Question Responses were analysed by summation for each participant, for each question for each participant and each survey for each participant (Section 6.1).
**Goal 2** To understand the distribution of behavioural archetypes within a population	
**Questions** Are all behavioural archetypes equally likely to be chosen? Are behavioural archetypes related to gender?	**Metrics** Chi-Square Test of Behavioural Archetype according to frequency and gender (Section 6.2).
**Goal 3** To understand whether the behavioural archetypes are related to emotional experience and psychological states	
**Questions** What are the differences (if any) in how respective behavioural archetypes relate to positive and negative emotions as well as psychological states?	**Metrics** Descriptive Statistics for each Behavioural Archetype relative to survey questions pertaining to positive emotions, negative emotions, and psychological states (Section 6.3).
**Goal 4** Do the behavioural archetypes possess internal validity?	
**Questions** Do the participants respond affirmatively to questions pertaining to key characteristics of the behavioural archetype? Must participants respond to all questions affirmatively to be considered exponents of that behavioural archetype? To what extent does the continuum of human emotion influence variation to closed response questions? Is there evidence that any of the behavioural archetypes are significantly more or less valid than others? Were other dependent variables predictors of internal validity for the behavioural archetype?	**Metrics** The first five questions for each Behavioural Archetype were specifically designed to elicit this information. It was, therefore, possible to validate the extent to which each participant’s behaviours corresponded to key characteristics of the behavioural archetype. Statistics relating to response variation were found and analysed. Internal validity scores were found to be normally distributed and homogeneity of variance tests were undertaken. Subsequently, ANOVA was performed to identify any predictors of internal validity (Section 6.4).
**Goal 5** Are the behavioural archetypes reliable?	
**Questions** Do the participants respond consistently? (With regard to questions pertaining to key characteristics of the behavioural archetype.)	**Metrics** The sum of participants’ internal validity scores for each Behavioural Archetype was found for each of five days of the study. These were plotted on a time series graph to enable any obvious visual trends to be seen. An essentially horizontal line is indicative of the stability of each behavioural archetype (Section 6.5).

**Table 11:** The Process of data transfer to binary input.

Digital Response Equivalent	Questionnaire Response Data
0	Not Felt
1	Felt
99	No Response

**Table 12:** Descriptive of the diary survey questions.

Behavioural Archetype	*N*#	Total Diary Survey Questions	Internal Validity Questions	Positive Emotion Questions	Negative Emotion Questions	Psychological States Questions
**Secure**	1	17	5	3	4	5
**Intimacy**	2	20	5	4	5	6
**Escapism**	3	15	5	2	4	4
**Narcissism**	4	17	5	3	4	5
**Discrepancy**	5	14	5	2	3	4

**Table 13:** Participants summary by behavioural archetype and gender.

Behavioural Archetype						
Gender	Secure	Intimacy	Escapism	Narcissism	Discrepancy	Total
Male	13	3	2	5	3	26
Female	5	8	2	1	3	19
**Total**	18	11	4	6	6	45

**Table 14:** Participants split by age group and gender.

Age Group	Male	Female	Total
18–24	3	10	13
25–34	16	7	23
35–44	7	2	9
**Total**	26	19	45

**Table 15:** Chi-Square value for equal proportions of participants by behavioural archetype and gender.

Statistics Test	Archetype Number	Participants Gender
**Chi-squared**	11.897	0.641
**Degree of Freedom**	4	1
**Asymptotic Significance**	0.018	0.423

**Table 16:** Behavioural archetype descriptions.

Behavioural Archetype (*n*)	Descriptive Statistic	Internal Characteristics Variable	Positive Emotions Variable	Negative Emotions Variable	Psychological States Variable
Secure (18)	Mean	0.6452	0.6210	0.3565	0.3356
	*N*	18	18	18	18
	Standard Deviation	0.16610	0.26943	0.24031	0.22968
	Median	0.7000	0.6333	0.3250	0.2933
	Minimum	0.33	0.18	0.03	0.07
	Maximum	0.84	1.00	1.00	1.00
	Range	0.51	0.82	0.97	0.93
Intimacy (11)	Mean	0.6485	0.7010	0.3697	0.4737
	*N*	11	11	11	11
	Standard Deviation	0.25794	0.26562	0.16293	0.25010
	Median	0.6933	0.6667	0.3333	0.3333
	Minimum	0.19	0.33	0.08	0.12
	Maximum	1.00	1.00	0.67	0.83
	Range	0.81	0.67	0.59	0.71
Escapism (4)	Mean	0.6833	0.8917	0.6667	0.6792
	*N*	4	4	4	4
	Standard Deviation	0.06289	0.11345	0.26105	0.11003
	Median	0.6600	0.9167	0.6333	0.6833
	Minimum	0.64	0.73	0.40	0.55
	Maximum	0.77	1.00	1.00	0.80
	Range	0.13	0.27	0.60	0.25
Narcissism (6)	Mean	0.6667	0.7074	0.3389	0.3756
	*N*	6	6	6	6
	Standard Deviation	0.23491	0.23409	0.15117	0.10342
	Median	0.6067	0.7444	0.3333	0.3733
	Minimum	0.33	0.42	0.18	0.23
	Maximum	1.00	1.00	0.50	0.53
	Range	0.67	0.58	0.32	0.31
Discrepancy (6)	Mean	0.5778	0.9222	0.5148	0.5083
	*N*	6	6	6	6
	Standard Deviation	0.28154	0.17470	0.21288	0.27543
	Median	0.5267	1.0000	0.5778	0.4167
	Minimum	0.25	0.57	0.20	0.25
	Maximum	1.00	1.00	0.73	0.95
	Range	0.75	0.43	0.53	0.70
Total (45)	Mean	0.6433	0.7163	0.4060	0.4282
	*N*	45	45	45	45
	Standard Deviation	0.20529	0.25888	0.22467	0.23721
	Median	0.6400	0.7111	0.3500	0.3333
	Minimum	0.19	0.18	0.03	0.07
	Maximum	1.00	1.00	1.00	1.00
	Range	0.81	0.82	0.97	0.93

**Table 17:** Mean internal characteristic validity measure by gender.

Gender	Mean	*N*	Standard Deviation	Median	Minimum	Maximum	Range
Male	0.6472	26	0.21981	0.6800	0.19	1.00	0.81
Female	0.6379	19	0.18936	0.6400	0.36	1.00	0.64
Total	0.6433	45	0.20529	0.6400	0.19	1.00	0.81

**Table 18:** Mean internal characteristic validity measure by age group.

Age Group	Mean	*N*	Standard Deviation	Median	Minimum	Maximum	Range
18–24	0.5774	13	0.17018	0.5600	0.25	0.80	0.55
25–34	0.6545	23	0.23858	0.6800	0.19	1.00	0.81
35–44	0.7096	9	0.13949	0.6800	0.53	1.00	0.47
Total	0.6433	45	0.20529	0.6400	0.19	1.00	0.81

**Table 19:** Mean internal characteristic validity measure by behavioural archetype.

Behavioural Archetype	Mean	*N*	Standard Deviation	Median	Minimum	Maximum	Range
Secure	0.6452	18	0.16610	0.7000	0.33	0.84	0.51
Intimate	0.6485	11	0.25794	0.6933	0.19	1.00	0.81
Escapist	0.6833	4	0.06289	0.6600	0.64	0.77	0.13
Narcissist	0.6667	6	0.23491	0.6067	0.33	1.00	0.67
Discrepancy	0.5778	6	0.28154	0.5267	0.25	1.00	0.75
Total	0.6433	45	0.20529	0.6400	0.19	1.00	0.81

**Table 20:** Mean scores by day and behavioural archetype.

5 Study Days	Secure	Intimacy	Escapism	Narcissism	Discrepancy
D1 Mean	24.5	29	34.25	24.33	24.4
D2 Mean	25.5	28.5	30.5	27.17	25.6
D3 Mean	22.44	28.13	31.5	27.5	26.6
D4 Mean	21.75	23.5	30.75	27.17	24.6
D5 Mean	20.63	25.88	32.75	24.17	27.4
Overall Mean	22.964	27.002	31.95	26.068	25.72

**Figure A1:**
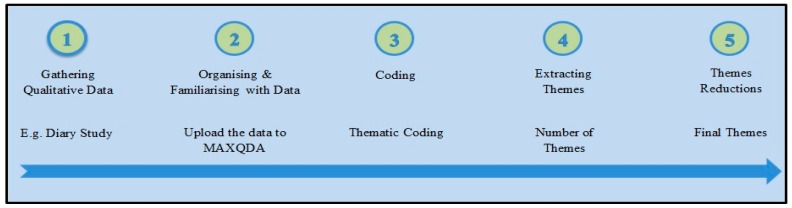
Coding stages.

## Appendix A

The first section of this article describes an exploratory study and the preliminary findings of a validation study. The sample for the first study consisted of 18 participants and was a convenience sample. Table A1 shows demographic information about the participants.

**Table A1:** Demographics of the participants.

Demographic Variable		No. of Participants
Gender	Male	9
	Female	9
Age in years	18–24	9
	25–34	8
	35–44	1
	45 or older	-

### Appendix A.1. Exploratory Study: Analysis

The theoretical framework for this research was based on the theoretical position of Braun and Clarke [60]. A qualitative diary study was the main method employed to obtain data on individuals’ daily interactions via social media. The diary notes were subjected to thematic analysis, an approach that is generally employed to assess qualitative data by uncovering the themes and patterns they contain. This approach facilitates the assessment of the data in two primary ways. First, using inductive coding, and second, by determining the consistency and sufficiency of the information that is relevant to the research questions. The main method used to mark themes during the process is to highlight any sentences or words in the data set that convey particular ideas that are connected to the research problem and to classify them according to their meanings or patterns, as per Braun and Clarke (ibid).

The diary entries were assessed using a version of the three-level process recommended by [103], which involved coding the data and organising it into themes. According to Braun and Clarke (ibid), the patterns in a data set can be recognised through a process of revision, data coding, data familiarisation, and theme development. Due to the volume of information contained in the diary entries, we used statistical analysis software to assess the data efficiently and systematically. Table A2 illustrates the thematic analysis process, which was following the recommendations of Braun and Clarke (ibid).

**Table A2:** Example of the thematic analysis.

Data Extract	Code
At the moment I am really excited and happy because my number of followers increased and my timeline is active	Feeling Reputation

The first stage of the analysis involved assigning the diary notes collected via Evernote to individual participant folders and then reading the data a number of times, in order to identify potential themes. After this, the diary notes were imported into the MAXQDA software (VERBI Software GmbH, Berlin, Germany), and the raw data were encoded, sentence by sentence, and line by line using the themes identified in the first stage (see Table A2 above). Any information relating to these potential themes was highlighted using quantitative coding, and a further assessment of the data was carried out in order to combine the themes into categories. The analysis generated the following 13 preliminary categories: Role, Online identity, Technical issues, Identity ruining, Drive, Online profile identification, Features, Social media platforms, Motivation, Emotional state, Abnormal behaviour, Context, and Online attachment style.

In the next stage of the analysis, the categories generated were prioritised and interpreted, and subsequently condensed. The five stages of the analysis described above are illustrated in Figure A1.

### Appendix A.2. Validation Study

The goal of this study was to verify the validity of the data collected previously. To achieve this, the data set was scrutinised using focus group sessions and a closed-card sorting technique. Fourteen participants were recruited for the card sorting and focus group study. Six participants were selected from the first phase sample to avoid analytical bias and increase the credibility of the findings from their perspectives. This also served as a member checking [104] technique—a technique for validating qualitative study findings. The other eight participants were recruited from a different cohort following the same recruitment procedure in the exploration study.

Closed-card sorting was used to refine the findings of the first phase. This card sorting technique is useful for gaining insight into participants’ perspective on findings and how they are related to each other, with discussion and observation being used to obtain additional feedback. The participants were given the pre-defined primary groups, pens, notes, and cards. The cards related to every category index concluded from the analysis of the first phase, for instance, psychological states and features. Eight people participated in the first card-sorting session; they were split into two teams and asked to discuss and sort cards within their teams, then discuss the cards with the other team. We also conducted one-to-one interviews to obtain insight into individual participants’ views. This is another way of demonstrating validity and avoiding groupthink [105]. Participants were told to provide the cards and refine the groups, as well as the concepts of every category from the findings, together with the pre-defined cards. Participants were encouraged to think aloud as they sorted, and to describe their reasons for arranging the cards in a particular way, as well as any uncertainties they had about where to place cards or relationships between groups of cards. After completing the card-sorting task, the participants were required to answer some questions about the sorting exercise. A spreadsheet was used to analyse the closed card sort data.
